# 257. Shared antibody sequences reveal novel public clonotypes of antibodies within and between HIV infected and healthy individuals

**DOI:** 10.1093/ofid/ofad500.329

**Published:** 2023-11-27

**Authors:** Arthur Chang, Mark D Hicar

**Affiliations:** University of Nebraska Medical Center, Omaha, Nebraska; University at Buffalo, Buffalo, New York

## Abstract

**Background:**

Public clonotypes, antibodies against specific antigens in unrelated individuals that have genetic similarities, have been shown in a variety of infections, including SARS-CoV-2 and HIV. These can be exploited as potential diagnostics and to identify past infections that may be related to post-infectious disorders, such as Kawasaki disease, Multiple Sclerosis or Alzheimer's disease.

**Methods:**

Heavy chain variable sequences were retrieved from public biorepositories (Bioprojects PRJNA486667, PRJNA638224,PRJNA527941) from 270 subjects. These were derived from labeled cohorts: Healthy (116), HIV broadly neutralizing (46), HIV non-broadly-neutralizing (50), HIV-negative (43), and COVID-19 (15). We utilized the Immcantation package of software run on our SUNY Buffalo computational cluster. After PRESTo annotation, duplicate sequences were collapsed and sequences of only single counts were removed. Clonal groups were determined using SCOPer requiring IGHV, IGHJ, and CDR3 amino acid sequence to be perfectly matched. Figures and statistics were generated with immcantation, excel, pandas, and graphpad prism 8.

**Results:**

There were 5.5 million unique heavy chain sequences from 32 million sequences. Defining public clonotypes as completely sharing CDR3 amino acid sequence and having predicted same germline variable, D, and J chains, 6861 (0.12%) shared clones were found. The distributions of CDR3 lengths showed skewing to shorter CDR3s (mean length of 10.5). There were no stringently defined clones noted in all members. Within HIV infected persons the two most shared clones both used VH1-18, JH4, had CDRaa 8, and were immature IGM/D. Three clones were used in near 10% of cases across both non-HIV and HIV datasets with prominent usage of VH 4 family members and were less mutated and IGM/D. There were eight clones solely shared > 10% within non-HIV/non-Covid infected individuals, with the top shared clones all consistent with matured IGG responses with prominent predicted use of VH3-7 and a number of novel CDR3s noted.

**Figure:**
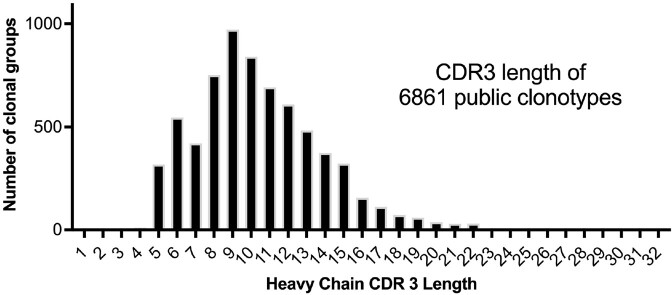

Predicted CDR3 length using incantation was compared for cohort of clones that shared predicted V, D, J usage and the exact CDR3 amino acids between individuals. This distribution failed normality testing.

**Conclusion:**

Identification of public clonotypes will show stochastic bias toward shorter CDR3 regions. There were differences between HIV and non-HIV cohorts and a number of novel public clonotypes were found.

**Disclosures:**

**Mark D. Hicar, MD/PhD**, Pfizer: site investigator for 2 trial

